# *catena*-Poly[[{2-meth­oxy-6-[(4-methyl­phen­yl)imino­meth­yl]phenolato}­cad­mium(II)]-di-μ_2_-chlorido-[dimethanol­cadmium(II)]-di-μ_2_-chlorido-[{2-meth­oxy-6-[(4-methyl­phen­yl)imino­meth­yl]­phenolato}cadmium(II)]-di-μ_2_-chlorido]

**DOI:** 10.1107/S1600536808033527

**Published:** 2008-10-18

**Authors:** Hui-Duo Xian, Hua-Qiong Li, Jian-Feng Liu, Guo-Liang Zhao

**Affiliations:** a Zhejiang Key Laboratory for Reactive Chemistry on Solid Surfaces, Institute of Physical Chemistry, Zhejiang Normal University, Jinhua, Zhejiang 321004, People’s Republic of China, and College of Chemistry and Life Science, Zhejiang Normal University, Jinhua 321004, Zhejiang, People’s Republic of China

## Abstract

The structure of the title compound, [Cd_3_Cl_6_(C_15_H_15_NO_2_)_2_(CH_4_O)_2_]_*n*_, is based on a layered zigzag polymeric chain along the *c* axis. The Cd^II^ ions are linked by double chlorine bridges alternating between one CdCl_4_(CH_3_OH)_2_ and two CdCl_4_(C_15_H_15_NO_2_) octa­hedral coordination units. Additional intrachain N—H⋯O and O—H⋯Cl hydrogen-bond interactions stabilize this arrangement.

## Related literature

For related literature, see: Henkel & Krebs (2004 ▶); Suen & Wang (2007 ▶); Wang *et al.* (2005 ▶); Zhang & Bu (2008 ▶); De Girolamo *et al.* (2007 ▶).
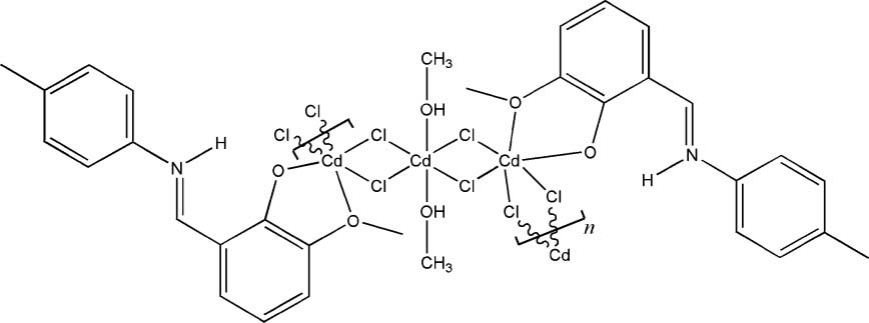


## Experimental

### 

#### Crystal data

[Cd_3_Cl_6_(C_15_H_15_NO_2_)_2_(CH_4_O)_2_]
*M* *_r_* = 1096.57
Monoclinic, 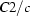

*a* = 19.7697 (5) Å
*b* = 13.9554 (3) Å
*c* = 15.1449 (4) Å
β = 110.4230 (10)°
*V* = 3915.74 (17) Å^3^
*Z* = 4
Mo *K*α radiation
μ = 2.07 mm^−1^
*T* = 296 (2) K
0.15 × 0.13 × 0.05 mm

#### Data collection

Bruker APEXII area-detector diffractometer
Absorption correction: multi-scan (*SADABS*; Sheldrick, 1996 ▶) *T*_min_ = 0.736, *T*_max_ = 0.898
13592 measured reflections
3350 independent reflections
2773 reflections with *I* > 2σ(*I*)
*R*_int_ = 0.033

#### Refinement

*R*[*F* ^2^ > 2σ(*F* ^2^)] = 0.026
*wR*(*F* ^2^) = 0.060
*S* = 1.05
3350 reflections
226 parameters
1 restraint
H atoms treated by a mixture of independent and constrained refinement
Δρ_max_ = 0.36 e Å^−3^
Δρ_min_ = −0.34 e Å^−3^

### 

Data collection: *APEX2* (Bruker, 2006 ▶); cell refinement: *SAINT* (Bruker, 2006 ▶); data reduction: *SAINT*; program(s) used to solve structure: *SHELXS97* (Sheldrick, 2008 ▶); program(s) used to refine structure: *SHELXL97* (Sheldrick, 2008 ▶); molecular graphics: *SHELXTL* (Sheldrick, 2008 ▶); software used to prepare material for publication: *SHELXL97* (Sheldrick, 2008 ▶).

## Supplementary Material

Crystal structure: contains datablocks I, global. DOI: 10.1107/S1600536808033527/at2645sup1.cif

Structure factors: contains datablocks I. DOI: 10.1107/S1600536808033527/at2645Isup2.hkl

Additional supplementary materials:  crystallographic information; 3D view; checkCIF report

## Figures and Tables

**Table 1:** Hydrogen-bond geometry (Å, °)

*D*—H⋯*A*	*D*—H	H⋯*A*	*D*⋯*A*	*D*—H⋯*A*
N1—H1*D*⋯O1	0.86	1.88	2.574 (3)	137
O3—H3*C*⋯Cl3	0.838 (19)	2.38 (2)	3.213 (3)	170 (5)

**Fig. 1.**
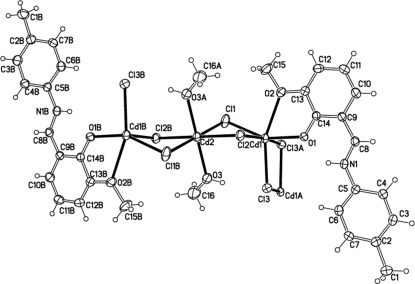
The structure of title compound, with displacement ellipsoids for non-H atoms drawn at the 50% probability level.

## supplementary crystallographic information

### Comment

There has been an increasing interest in the coordination chemistry of cadmium in recent years due to the increased recognition of its role in biological organisms (Henkel & Krebs, 2004), as well as in molecular-based materials (De Girolamo *et al.*, 2007). In the quest for molecular-based materials with interesting properties, much attention has been given to one-, two- and three-dimensional extended solids which involve cadmium (Suen & Wang, 2007; Wang *et al.*, 2005; Zhang & Bu, 2008). Complexes of the type Cd*X*_2_ (*X* = CI or Br) with organic bases typically form one- or two-dimensional halogen-bridged chain compounds with six-coordination octahedral cadmium(II). Here, we describe the synthesis and crystal structure of the cadmium(II) chloride complex with 2-[(4-methylphenylimino)methyl]-6-methoxyphenol.

The crystal structure of the title compound (I) has features of the monoclinic space group *C*2/*c*. As illustrated in Fig. 1, the structure comprises an alternating polymeric chain layer along the *c* axis. The Cd^II^ ions are linked into an infinite chain by double chlorine bridges, The Cd(1)···Cd(2) and Cd(1)···Cd(1A) distances in the molecule are 3.7087 (3) and 3.8756 (4) Å, respectively.

### Experimental

A solution of CdCl_2_ (2 mmol) in methanol (20 ml) was added to a methanol solution (20 ml) of the Schiff base ligand (2 mmol, 0.48 g). Red crystals of (I) were isolated after two weeks.

### Refinement

The H atoms bonded to C and N atoms were positioned geometrically and refined using a riding model [aromatic C—H 0.93 Å, methylic C—H = 0.96 Å and N—H = 0.86 Å, *U*_iso_(H) = 1.2 or 1.5*U*_eq_(C, N)]. The H atoms bonded to O atoms were located in a difference Fourier maps and refined with O—H distance restraints of 0.85 (2) and *U*_iso_(H) = 1.5*U*_eq_(O).

### Crystal data

**Table:**

[Cd_3_Cl_6_(C_15_H_15_NO_2_)_2_(CH_4_O)_2_]	*F*(000) = 2152
*M**_r_* = 1096.57	*D*_x_ = 1.860 Mg m^−^^3^
Monoclinic, *C*2/*c*	Mo *K*α radiation, λ = 0.71073 Å
Hall symbol: -C 2yc	Cell parameters from 3942 reflections
*a* = 19.7697 (5) Å	θ = 1.8–25.0°
*b* = 13.9554 (3) Å	µ = 2.07 mm^−^^1^
*c* = 15.1449 (4) Å	*T* = 296 K
β = 110.423 (1)°	Block, red
*V* = 3915.74 (17) Å^3^	0.15 × 0.13 × 0.05 mm
*Z* = 4	

### Data collection

**Table:**

Bruker APEXII diffractometer	3350 independent reflections
Radiation source: fine-focus sealed tube	2773 reflections with *I* > 2σ(*I*)
graphite	*R*_int_ = 0.033
ω scans	θ_max_ = 25.0°, θ_min_ = 1.8°
Absorption correction: multi-scan (SADABS; Sheldrick, 1996)	*h* = −23→23
*T*_min_ = 0.736, *T*_max_ = 0.898	*k* = −16→16
13592 measured reflections	*l* = −15→18

### Refinement

**Table:**

Refinement on *F*^2^	Primary atom site location: structure-invariant direct methods
Least-squares matrix: full	Secondary atom site location: difference Fourier map
*R*[*F*^2^ > 2σ(*F*^2^)] = 0.026	Hydrogen site location: inferred from neighbouring sites
*wR*(*F*^2^) = 0.060	H atoms treated by a mixture of independent and constrained refinement
*S* = 1.05	*w* = 1/[σ^2^(*F*_o_^2^) + (0.0247*P*)^2^ + 2.9063*P*] where *P* = (*F*_o_^2^ + 2*F*_c_^2^)/3
3350 reflections	(Δ/σ)_max_ = 0.002
226 parameters	Δρ_max_ = 0.36 e Å^−^^3^
1 restraint	Δρ_min_ = −0.34 e Å^−^^3^

### Special details

**Table:**

Geometry. All e.s.d.'s (except the e.s.d. in the dihedral angle between two l.s. planes) are estimated using the full covariance matrix. The cell e.s.d.'s are taken into account individually in the estimation of e.s.d.'s in distances, angles and torsion angles; correlations between e.s.d.'s in cell parameters are only used when they are defined by crystal symmetry. An approximate (isotropic) treatment of cell e.s.d.'s is used for estimating e.s.d.'s involving l.s. planes.
Refinement. Refinement of *F*^2^ against ALL reflections. The weighted *R*-factor *wR* and goodness of fit *S* are based on *F*^2^, conventional *R*-factors *R* are based on *F*, with *F* set to zero for negative *F*^2^. The threshold expression of *F*^2^ > σ(*F*^2^) is used only for calculating *R*-factors(gt) *etc*. and is not relevant to the choice of reflections for refinement. *R*-factors based on *F*^2^ are statistically about twice as large as those based on *F*, and *R*- factors based on ALL data will be even larger.

### Fractional atomic coordinates and isotropic or equivalent isotropic displacement parameters (Å^2^)

**Table:**

	*x*	*y*	*z*	*U*_iso_*/*U*_eq_	
Cd1	0.542829 (14)	1.284415 (15)	−0.113758 (18)	0.04061 (9)	
Cd2	0.5000	1.5000	0.0000	0.04260 (11)	
N1	0.46501 (15)	0.97593 (18)	−0.16754 (19)	0.0373 (6)	
H1D	0.4710	1.0367	−0.1704	0.045*	
O1	0.53998 (12)	1.12806 (14)	−0.10453 (16)	0.0440 (6)	
O2	0.65586 (13)	1.21264 (15)	0.01463 (18)	0.0502 (6)	
O3	0.41084 (17)	1.48567 (18)	−0.1495 (2)	0.0734 (9)	
H3C	0.406 (3)	1.431 (2)	−0.174 (3)	0.110*	
Cl1	0.59671 (6)	1.44929 (6)	−0.07253 (8)	0.0632 (3)	
Cl2	0.48990 (5)	1.31686 (6)	0.02126 (6)	0.0447 (2)	
Cl3	0.41178 (5)	1.27831 (6)	−0.24124 (6)	0.0449 (2)	
C1	0.2053 (2)	0.8336 (3)	−0.4388 (3)	0.0702 (12)	
H1A	0.2054	0.7648	−0.4396	0.105*	
H1B	0.1995	0.8574	−0.5005	0.105*	
H1C	0.1661	0.8558	−0.4206	0.105*	
C2	0.27528 (19)	0.8692 (2)	−0.3696 (3)	0.0470 (9)	
C3	0.3294 (2)	0.8076 (2)	−0.3173 (3)	0.0534 (10)	
H3A	0.3228	0.7420	−0.3272	0.064*	
C4	0.3923 (2)	0.8400 (2)	−0.2515 (3)	0.0487 (9)	
H4A	0.4278	0.7968	−0.2177	0.058*	
C5	0.40256 (18)	0.9373 (2)	−0.2360 (2)	0.0361 (8)	
C6	0.3509 (2)	1.0007 (2)	−0.2894 (3)	0.0433 (8)	
H6A	0.3586	1.0664	−0.2814	0.052*	
C7	0.28796 (19)	0.9666 (2)	−0.3544 (3)	0.0469 (9)	
H7A	0.2530	1.0098	−0.3891	0.056*	
C8	0.51477 (18)	0.9305 (2)	−0.1005 (2)	0.0373 (8)	
H8A	0.5095	0.8648	−0.0947	0.045*	
C9	0.57599 (18)	0.9754 (2)	−0.0364 (2)	0.0356 (8)	
C10	0.6274 (2)	0.9190 (2)	0.0325 (3)	0.0498 (9)	
H10A	0.6196	0.8537	0.0365	0.060*	
C11	0.6876 (2)	0.9600 (3)	0.0925 (3)	0.0557 (10)	
H11A	0.7218	0.9222	0.1367	0.067*	
C12	0.6997 (2)	1.0591 (3)	0.0893 (3)	0.0510 (9)	
H12A	0.7414	1.0863	0.1315	0.061*	
C13	0.65035 (18)	1.1156 (2)	0.0245 (2)	0.0398 (8)	
C14	0.58669 (17)	1.0755 (2)	−0.0415 (2)	0.0344 (7)	
C15	0.7108 (2)	1.2622 (3)	0.0885 (3)	0.0693 (12)	
H15A	0.7460	1.2170	0.1252	0.104*	
H15B	0.6893	1.2942	0.1282	0.104*	
H15C	0.7338	1.3084	0.0614	0.104*	
C16	0.3865 (3)	1.5562 (4)	−0.2154 (3)	0.110 (2)	
H16A	0.3495	1.5309	−0.2701	0.165*	
H16B	0.4257	1.5796	−0.2329	0.165*	
H16C	0.3670	1.6078	−0.1897	0.165*	

### Atomic displacement parameters (Å^2^)

**Table:**

	*U*^11^	*U*^22^	*U*^33^	*U*^12^	*U*^13^	*U*^23^
Cd1	0.04572 (18)	0.02655 (13)	0.04443 (16)	−0.00188 (10)	0.00927 (13)	−0.00170 (10)
Cd2	0.0499 (3)	0.02725 (18)	0.0422 (2)	0.00221 (15)	0.00547 (19)	−0.00150 (14)
N1	0.0407 (18)	0.0263 (13)	0.0430 (17)	−0.0029 (12)	0.0120 (15)	−0.0001 (12)
O1	0.0432 (15)	0.0273 (11)	0.0472 (14)	0.0025 (10)	−0.0022 (12)	0.0032 (10)
O2	0.0454 (16)	0.0383 (13)	0.0571 (16)	−0.0100 (11)	0.0055 (13)	−0.0043 (11)
O3	0.086 (2)	0.0433 (16)	0.0607 (19)	0.0093 (15)	−0.0132 (17)	−0.0099 (14)
Cl1	0.0737 (7)	0.0389 (5)	0.0889 (8)	−0.0198 (5)	0.0433 (6)	−0.0183 (5)
Cl2	0.0541 (6)	0.0300 (4)	0.0472 (5)	−0.0023 (4)	0.0141 (5)	0.0016 (4)
Cl3	0.0422 (5)	0.0437 (5)	0.0436 (5)	−0.0017 (4)	0.0085 (4)	−0.0031 (4)
C1	0.044 (3)	0.060 (3)	0.087 (3)	−0.001 (2)	−0.002 (2)	−0.008 (2)
C2	0.039 (2)	0.045 (2)	0.053 (2)	−0.0029 (17)	0.0115 (19)	−0.0053 (17)
C3	0.054 (3)	0.0310 (18)	0.065 (3)	−0.0059 (17)	0.009 (2)	−0.0031 (17)
C4	0.047 (2)	0.0344 (19)	0.052 (2)	0.0025 (16)	0.002 (2)	0.0024 (16)
C5	0.036 (2)	0.0332 (17)	0.0384 (19)	−0.0032 (14)	0.0118 (17)	−0.0014 (14)
C6	0.048 (2)	0.0312 (17)	0.048 (2)	−0.0007 (16)	0.0138 (19)	0.0014 (15)
C7	0.038 (2)	0.0418 (19)	0.052 (2)	0.0071 (16)	0.005 (2)	0.0014 (17)
C8	0.043 (2)	0.0285 (16)	0.039 (2)	0.0009 (15)	0.0134 (18)	0.0027 (14)
C9	0.038 (2)	0.0323 (16)	0.0347 (19)	0.0039 (14)	0.0108 (17)	0.0013 (14)
C10	0.059 (3)	0.0365 (19)	0.047 (2)	0.0086 (18)	0.009 (2)	0.0056 (16)
C11	0.055 (3)	0.048 (2)	0.048 (2)	0.0139 (19)	−0.002 (2)	0.0068 (18)
C12	0.040 (2)	0.058 (2)	0.044 (2)	0.0038 (18)	0.0008 (19)	−0.0074 (18)
C13	0.040 (2)	0.0357 (18)	0.040 (2)	−0.0002 (15)	0.0102 (18)	−0.0027 (15)
C14	0.037 (2)	0.0319 (17)	0.0341 (18)	0.0030 (14)	0.0121 (17)	−0.0015 (14)
C15	0.069 (3)	0.059 (2)	0.066 (3)	−0.026 (2)	0.005 (2)	−0.016 (2)
C16	0.148 (6)	0.076 (3)	0.062 (3)	0.018 (3)	−0.018 (3)	−0.001 (3)

### Geometric parameters (Å, °)

**Table:**

Cd1—O1	2.188 (2)	C2—C7	1.387 (5)
Cd1—Cl1	2.5208 (9)	C3—C4	1.371 (5)
Cd1—O2	2.597 (2)	C3—H3A	0.9300
Cd1—Cl3	2.6374 (9)	C4—C5	1.382 (4)
Cd1—Cl2	2.6410 (9)	C4—H4A	0.9300
Cd1—Cl3^i^	2.6476 (9)	C5—C6	1.380 (4)
Cd2—O3^ii^	2.343 (3)	C6—C7	1.375 (5)
Cd2—O3	2.343 (3)	C6—H6A	0.9300
Cd2—Cl2	2.5924 (8)	C7—H7A	0.9300
Cd2—Cl2^ii^	2.5924 (8)	C8—C9	1.407 (4)
Cd2—Cl1	2.6133 (10)	C8—H8A	0.9300
Cd2—Cl1^ii^	2.6133 (10)	C9—C10	1.414 (4)
N1—C8	1.306 (4)	C9—C14	1.418 (4)
N1—C5	1.413 (4)	C10—C11	1.348 (5)
N1—H1D	0.8600	C10—H10A	0.9300
O1—C14	1.299 (4)	C11—C12	1.408 (5)
O2—C13	1.372 (4)	C11—H11A	0.9300
O2—C15	1.435 (4)	C12—C13	1.366 (5)
O3—C16	1.365 (5)	C12—H12A	0.9300
O3—H3C	0.838 (19)	C13—C14	1.421 (4)
Cl3—Cd1^i^	2.6476 (9)	C15—H15A	0.9600
C1—C2	1.499 (5)	C15—H15B	0.9600
C1—H1A	0.9600	C15—H15C	0.9600
C1—H1B	0.9600	C16—H16A	0.9600
C1—H1C	0.9600	C16—H16B	0.9600
C2—C3	1.385 (5)	C16—H16C	0.9600
			
O1—Cd1—Cl1	155.83 (6)	C3—C2—C1	122.3 (3)
O1—Cd1—O2	66.56 (7)	C7—C2—C1	120.7 (3)
Cl1—Cd1—O2	89.30 (5)	C4—C3—C2	122.4 (3)
O1—Cd1—Cl3	88.51 (6)	C4—C3—H3A	118.8
Cl1—Cd1—Cl3	115.66 (3)	C2—C3—H3A	118.8
O2—Cd1—Cl3	154.74 (5)	C3—C4—C5	119.3 (3)
O1—Cd1—Cl2	95.48 (6)	C3—C4—H4A	120.3
Cl1—Cd1—Cl2	84.21 (3)	C5—C4—H4A	120.3
O2—Cd1—Cl2	87.50 (6)	C6—C5—C4	119.7 (3)
Cl3—Cd1—Cl2	91.09 (3)	C6—C5—N1	117.7 (3)
O1—Cd1—Cl3^i^	92.56 (6)	C4—C5—N1	122.6 (3)
Cl1—Cd1—Cl3^i^	89.92 (3)	C7—C6—C5	119.8 (3)
O2—Cd1—Cl3^i^	99.00 (6)	C7—C6—H6A	120.1
Cl3—Cd1—Cl3^i^	85.55 (3)	C5—C6—H6A	120.1
Cl2—Cd1—Cl3^i^	171.21 (3)	C6—C7—C2	121.7 (3)
O3^ii^—Cd2—O3	180.00 (12)	C6—C7—H7A	119.2
O3^ii^—Cd2—Cl2	91.64 (7)	C2—C7—H7A	119.2
O3—Cd2—Cl2	88.36 (7)	N1—C8—C9	123.6 (3)
O3^ii^—Cd2—Cl2^ii^	88.36 (7)	N1—C8—H8A	118.2
O3—Cd2—Cl2^ii^	91.64 (7)	C9—C8—H8A	118.2
Cl2—Cd2—Cl2^ii^	180.000 (1)	C8—C9—C10	119.0 (3)
O3^ii^—Cd2—Cl1	90.88 (9)	C8—C9—C14	120.7 (3)
O3—Cd2—Cl1	89.12 (9)	C10—C9—C14	120.4 (3)
Cl2—Cd2—Cl1	83.37 (3)	C11—C10—C9	120.0 (3)
Cl2^ii^—Cd2—Cl1	96.63 (3)	C11—C10—H10A	120.0
O3^ii^—Cd2—Cl1^ii^	89.12 (9)	C9—C10—H10A	120.0
O3—Cd2—Cl1^ii^	90.88 (9)	C10—C11—C12	120.9 (3)
Cl2—Cd2—Cl1^ii^	96.63 (3)	C10—C11—H11A	119.5
Cl2^ii^—Cd2—Cl1^ii^	83.37 (3)	C12—C11—H11A	119.5
Cl1—Cd2—Cl1^ii^	180.000 (1)	C13—C12—C11	120.3 (3)
C8—N1—C5	127.9 (3)	C13—C12—H12A	119.9
C8—N1—H1D	116.1	C11—C12—H12A	119.9
C5—N1—H1D	116.1	C12—C13—O2	125.7 (3)
C14—O1—Cd1	125.63 (19)	C12—C13—C14	120.9 (3)
C13—O2—C15	117.3 (3)	O2—C13—C14	113.4 (3)
C13—O2—Cd1	112.48 (19)	O1—C14—C9	121.0 (3)
C15—O2—Cd1	128.1 (2)	O1—C14—C13	121.5 (3)
C16—O3—Cd2	126.9 (3)	C9—C14—C13	117.5 (3)
C16—O3—H3C	112 (4)	O2—C15—H15A	109.5
Cd2—O3—H3C	116 (4)	O2—C15—H15B	109.5
Cd1—Cl1—Cd2	92.48 (3)	H15A—C15—H15B	109.5
Cd2—Cl2—Cd1	90.25 (3)	O2—C15—H15C	109.5
Cd1—Cl3—Cd1^i^	94.33 (3)	H15A—C15—H15C	109.5
C2—C1—H1A	109.5	H15B—C15—H15C	109.5
C2—C1—H1B	109.5	O3—C16—H16A	109.5
H1A—C1—H1B	109.5	O3—C16—H16B	109.5
C2—C1—H1C	109.5	H16A—C16—H16B	109.5
H1A—C1—H1C	109.5	O3—C16—H16C	109.5
H1B—C1—H1C	109.5	H16A—C16—H16C	109.5
C3—C2—C7	117.0 (3)	H16B—C16—H16C	109.5
			
Cl1—Cd1—O1—C14	9.2 (3)	Cl2—Cd1—Cl3—Cd1^i^	168.17 (2)
O2—Cd1—O1—C14	6.0 (2)	Cl3^i^—Cd1—Cl3—Cd1^i^	−3.70 (4)
Cl3—Cd1—O1—C14	−169.9 (2)	C7—C2—C3—C4	1.6 (6)
Cl2—Cd1—O1—C14	−78.9 (2)	C1—C2—C3—C4	−177.5 (4)
Cl3^i^—Cd1—O1—C14	104.7 (2)	C2—C3—C4—C5	0.3 (6)
O1—Cd1—O2—C13	−5.0 (2)	C3—C4—C5—C6	−2.7 (5)
Cl1—Cd1—O2—C13	176.3 (2)	C3—C4—C5—N1	178.1 (3)
Cl3—Cd1—O2—C13	4.8 (3)	C8—N1—C5—C6	168.6 (3)
Cl2—Cd1—O2—C13	92.1 (2)	C8—N1—C5—C4	−12.3 (5)
Cl3^i^—Cd1—O2—C13	−93.9 (2)	C4—C5—C6—C7	3.3 (5)
O1—Cd1—O2—C15	−167.6 (3)	N1—C5—C6—C7	−177.6 (3)
Cl1—Cd1—O2—C15	13.8 (3)	C5—C6—C7—C2	−1.4 (5)
Cl3—Cd1—O2—C15	−157.7 (3)	C3—C2—C7—C6	−1.0 (5)
Cl2—Cd1—O2—C15	−70.5 (3)	C1—C2—C7—C6	178.1 (4)
Cl3^i^—Cd1—O2—C15	103.6 (3)	C5—N1—C8—C9	178.9 (3)
Cl2—Cd2—O3—C16	−174.9 (4)	N1—C8—C9—C10	−178.8 (3)
Cl2^ii^—Cd2—O3—C16	5.1 (4)	N1—C8—C9—C14	0.3 (5)
Cl1—Cd2—O3—C16	−91.5 (4)	C8—C9—C10—C11	177.7 (3)
Cl1^ii^—Cd2—O3—C16	88.5 (4)	C14—C9—C10—C11	−1.4 (5)
O1—Cd1—Cl1—Cd2	−113.74 (16)	C9—C10—C11—C12	1.6 (6)
O2—Cd1—Cl1—Cd2	−110.81 (6)	C10—C11—C12—C13	−0.4 (6)
Cl3—Cd1—Cl1—Cd2	65.17 (4)	C11—C12—C13—O2	179.8 (3)
Cl2—Cd1—Cl1—Cd2	−23.25 (3)	C11—C12—C13—C14	−0.9 (5)
Cl3^i^—Cd1—Cl1—Cd2	150.19 (3)	C15—O2—C13—C12	−12.1 (5)
O3^ii^—Cd2—Cl1—Cd1	115.31 (7)	Cd1—O2—C13—C12	−176.7 (3)
O3—Cd2—Cl1—Cd1	−64.69 (7)	C15—O2—C13—C14	168.6 (3)
Cl2—Cd2—Cl1—Cd1	23.75 (3)	Cd1—O2—C13—C14	4.0 (3)
Cl2^ii^—Cd2—Cl1—Cd1	−156.25 (3)	Cd1—O1—C14—C9	173.8 (2)
O3^ii^—Cd2—Cl2—Cd1	−113.28 (9)	Cd1—O1—C14—C13	−6.4 (4)
O3—Cd2—Cl2—Cd1	66.72 (9)	C8—C9—C14—O1	0.8 (5)
Cl1—Cd2—Cl2—Cd1	−22.59 (3)	C10—C9—C14—O1	179.9 (3)
Cl1^ii^—Cd2—Cl2—Cd1	157.41 (3)	C8—C9—C14—C13	−178.9 (3)
O1—Cd1—Cl2—Cd2	179.14 (6)	C10—C9—C14—C13	0.1 (5)
Cl1—Cd1—Cl2—Cd2	23.43 (3)	C12—C13—C14—O1	−178.8 (3)
O2—Cd1—Cl2—Cd2	112.98 (5)	O2—C13—C14—O1	0.6 (4)
O1—Cd1—Cl3—Cd1^i^	−96.38 (6)	C12—C13—C14—C9	1.0 (5)
Cl1—Cd1—Cl3—Cd1^i^	84.07 (3)	O2—C13—C14—C9	−179.6 (3)
O2—Cd1—Cl3—Cd1^i^	−105.40 (14)		

Symmetry codes: (i) −*x*+1, *y*, −*z*−1/2; (ii) −*x*+1, −*y*+3, −*z*.

### Hydrogen-bond geometry (Å, °)

**Table:**

*D*—H···*A*	*D*—H	H···*A*	*D*···*A*	*D*—H···*A*
N1—H1D···O1	0.86	1.88	2.574 (3)	137
O3—H3C···Cl3	0.84 (2)	2.38 (2)	3.213 (3)	170 (5)
